# Exploring the breastfeeding knowledge level and its influencing factors of pregnant women with gestational diabetes mellitus

**DOI:** 10.1186/s12884-020-03430-9

**Published:** 2020-11-23

**Authors:** Yan Wang, Hua-xuan You, Bi-ru Luo

**Affiliations:** 1 Key Laboratory of Birth Defects and Related Diseases of Women and Children (Sichuan University), Ministry of Education, West China Second University Hospital /West China School of Nursing, Sichuan University, Chengdu, 610041 China; 2 Department of Reproductive Endocrinology Nursing, West China Second University Hospital, Sichuan University/West China School of Nursing, Sichuan University, Chengdu, 610041 China; 3 Key Laboratory of Birth Defects and Related Diseases of Women and Children (Sichuan University), Ministry of Education, Chengdu, 610041 China; 4 Department of Nursing, West China Second University Hospital, Sichuan University, Chengdu, 610041 China

**Keywords:** Gestational diabetes mellitus, Breastfeeding, Knowledge

## Abstract

**Background:**

Studies reveal that promoting the breastfeeding knowledge level help to improve breastfeeding behaviors. Promoting breastfeeding knowledge is a simple and economical way to increase breastfeeding rates. However, there are no studies focus on the level of breastfeeding knowledge and factors influencing the knowledge in women with gestational diabetes mellitus (GDM), which is defined as any degree of glucose tolerance impairment first diagnosed during pregnancy. Thus, the objectives of this study were to investigate the breastfeeding knowledge level of GDM pregnant women and explore factors influencing the knowledge level.

**Methods:**

Cross-sectional survey and convenience sampling were conducted in this study. The sociodemographic characteristics, caregivers in pregnancy, knowledge source, breastfeeding status and breast status information of participants were collected. Breastfeeding Knowledge Scale was used to assess the breastfeeding knowledge level of pregnant women with GDM. Multiple linear regression was used to analyze the influence factors of breastfeeding knowledge level in this study.

**Results:**

A total of 226 questionnaires were issued and finally 212 valid questionnaires were collected. Some misconceptions still existed (e.g. ‘breastfeeding cannot prevent your baby from being overweight’ and ‘it is advisable to breastfeed 3-4 times per day within 2-3 days after delivery’), although women with GDM had a good score of breastfeeding knowledge (mean score: 103.5 ± 10.4). Multiple linear regression analysis found that gestational age, family per capita monthly income, educational level, knowledge source were the independent protective factors for breastfeeding knowledge and minority nationality was the independent risk factor. The educational level had the greatest influence on the breastfeeding knowledge level of GDM pregnant women (*β* = 0.210, *t* = 2.978, *P* = 0.003).

**Conclusion:**

GDM pregnant women with insufficient gestational age, low educational level, low family per capita monthly income and single access to knowledge should be included in the focus of health education on breastfeeding. In-depth and systematic health education should be conducted for pregnant women with GDM to improve their breastfeeding rate.

## Background

Breastfeeding is one of the measures proposed by the World Health Organization (WHO) and United Nation Children’s Fund (UNICEF) to protect the health of pregnant women and their children [1, 2]. Gestational diabetes mellitus (GDM) is defined as any degree of glucose intolerance with onset or first recognition during pregnancy [3]. GDM has adverse effects on the maternal and neonatal outcomes. A large number of studies have confirmed the short-term and long-term benefits of breastfeeding for pregnant women with GDM and their offspring [4]. Breastfeeding can help GDM patients regulate their weight and blood glucose levels and prevent the recurrence of GDM and type 2 diabetes after delivery. For the offspring of women with GDM, breastfeeding can help to reduce the risks of developing obesity and type 2 diabetes in adulthood [5, 6]. Therefore, breastfeeding is especially recommended and encouraged for women who have GDM.

Women with GDM should insist on breastfeeding. However, studies have shown that GDM women have less willingness to breastfeed and have lower breastfeeding rate than those of normal women at the same stage. Moreover, the breastfeeding rates of GDM women are falling faster [7–10]. Several studies have reported that women with GDM have lower rates of breastfeeding and earlier interruption of breastfeeding in the first 6 months [11, 12]. Study has shown that the main factors that hinder breastfeeding include maternal factors, social factors and work-related factors [13]. For example, giving birth by cesarean delivery [14], receiving a recommendation from health workers to formula-feed [14], without social support from husband and other relative [15], lack of empathy from female colleagues, uncaring attitudes to breastfeeding, discouragement and criticism from employers and colleagues, difficulty extending maternal leave [15, 16] negatively affect breastfeeding. Lack of knowledge is one of the most important barriers of breastfeeding [17]. On the contrary, higher breastfeeding knowledge level is significantly associated with greater breastfeeding intention [18], and stronger breastfeeding confidence [19]. Improving pregnant women’s breastfeeding knowledge level can effectively promote the occurrence of breastfeeding behavior and extend actual lactation duration [19–22].

However, current studies have shown that the breastfeeding knowledge level among pregnant women or mothers is not very ideal [23, 24]. Some misconceptions still existed, in spite of considerable awareness of the advantages of breastfeeding [23, 25]. The breastfeeding knowledge level is influenced many factors such as educational level and cultural beliefs [25]. A comprehensive understanding of the factors affecting the breastfeeding knowledge level is conducive to the development of targeted education to improve the knowledge level. However, there are few studies on the breastfeeding knowledge level and it influencing factors in pregnant women with GDM. Therefore, the objectives of this study were to investigate the breastfeeding knowledge level of pregnant women with GDM, and then explore the influencing factors on the knowledge level of breastfeeding.

## Methods

### Setting and participants

We conducted a cross-sectional survey in West China Second University Hospital, Sichuan University, which is a women and children’s medical center in West China serving > 5 provinces. The five provinces, including Sichuan, Guizhou, Yunnan, Qinghai and the Tibet Autonomous Region, lag behind in economic development and maternal and child health care in China. The objectives of this study were to investigate the breastfeeding knowledge level of pregnant women with GDM, and then explore the influencing factors on the knowledge level of breastfeeding. Convenience sampling was used in this study. We selected pregnant women who were waiting to birth from July to October 2017 in the Department of Obstetrics. Pregnant women of 18 years older and had a diagnosis of GDM were included in this survey. Pregnant women were excluded if they: had a history of type 1diabetes or type 2 diabetes; cannot read and write in Chinese; did not sign the informed consent.

Pregnant women with 2 h 75-g Oral Glucose Tolerance Test (OGTT) values exceeding established thresholds (fasting 5.1 mmol/L, 1 h 10.0 mmol/L, 2 h 8.5 mmol/L) were diagnosed as GDM.

### Data collection

We used self-designed questionnaire to collect sociodemographic information such as age, educational level, marital status, the number of childbirths, occupation, family per capita monthly income, nationality, and caregivers in pregnancy, breast status information such as history of breast surgery, breastfeeding status information such as breastfeeding experiences (see Additional file 1).

Breastfeeding Knowledge Scale [26] was used to assess pregnant women’s perception of breastfeeding. This scales was developed [27] and by later revised by Zhu, Y. and Wan, H [26].. Breastfeeding Knowledge Scale included 4 parts: benefits of exclusive breastfeeding, breastfeeding skills, breastfeeding storage methods and breastfeeding conditions. This scale was a Chinese 5-point Likert scale and with a total of 25 items. Responses range from “Very much agree” to “Very much disagree”. Breastfeeding knowledge scores ranged from 25 to 125, with higher scores indicating the higher breastfeeding knowledge level. Cronbach’s a coefficient is 0.820.

### Statistical analysis

The mean and standard deviation (*M ± SD*) were used to describe the quantitative data with normal distribution and approximately normal distribution. T-test and variance analysis were used for univariate analysis. Multiple linear stepwise regression analysis was used for multi-factor analysis (*a* = 0.05). Durbin Watson was used to test the independence of the independent variables. Collinearity diagnosis was considered in this study. SPSS23.0 (SPSS Inc., Chicago, IL) was used for statistical analysis.

## Results

### Characteristics of participants

A total of 226 questionnaires were issued and finally 212 valid questionnaires were recovered, with an effective recovery rate of 93.8%. Most pregnant women were between the age of 24 and 44 years old. 65.1% of participants had a bachelor’s degree or above, and 40.1% of participants had a family’s per capita monthly income of 5001–10,000 yuan. A total of 191 (90.1%) participants came from urban, and 21 (9.9%) participants came from rural. Husbands and mothers were the main caregivers of participants, accounting for 57.1 and 42.0%, respectively. The main sources of breastfeeding knowledge of pregnant women are family members and friends (55.2%), followed by medical staff (46.7%), books (44.8%), the Internet (44.3%), newspapers and magazines (12.3%) and television (10.8%) (Table 1).

**Table 1:** The characteristic of pregnant women

variable	Frequency (*n* = 212)	Percent (*%*)
Age (year)		
≤ 29	33	15.6
30–34	85	30.1
35–39	67	31.6
≥ 40	27	12.7
Gestational age (week)		
≤ 36^+ 6^	42	19.8
≥ 37	170	80.2
Multipara	111	52.2
Educational level		
Junior High and below	14	6.6
Senior High	15	7.1
Junior college	45	21.2
Bachelor or above	138	65.1
Occupation		
Professional	36	17.0
Administrative	62	29.2
Clerk	55	25.9
Farmer	2	0.9
Freelance	29	13.7
Unemployed	28	13.2
Nationality		
Han	203	95.8
Minority	9	4.2
Marital status		
Never married	0	0
Married	210	99.0
Divorced	2	1.0
Family per capita monthly income (yuan)		
< 3000	15	7.1
3001–5000	51	24.1
5001–10,000	91	42.9
> 10,000	55	25.9
Caregivers		
Pregnant women	41	19.3
Husband	121	57.1
Mother	89	42.0
Mother-in-law	35	16.5
Nanny	4	1.9
Relatives	7	3.3
Husband’s educational level		
Junior High and below	11	5.2
Senior High	12	5.7
Junior college	46	21.7
Bachelor or above	143	67.5
Knowledge source		
Book	95	44.8
Newspaper/magazine	26	12.3
Internet	94	44.3
Television programs	23	10.8
Family member or friends	117	55.2
Medical staff		
Number of knowledge source		
1	87	41.0
≥ 2	125	59.0
Breastfeeding experience		
Yes	99	46.2
No	113	53.8
Breastfeeding intention		
Yes	210	99.1
No	2	0.9
Health education on breastfeeding		
Yes	124	58.5
No	88	41.5

### Breastfeeding knowledge level of participants

The average total breastfeeding score of 212 participants was 103.5 ± 10.4 points, and the range of respondent scores was 62 points to 125 points. Pregnant women with GDM had a considerable awareness of some breastfeeding knowledge such as ‘exclusive breastfeeding is the best option for baby up to 6 months of age’, and ‘containing antibody composition in breast milk, can enhance baby immunity and reduce disease occurrence’. However, some misconceptions (e.g. ‘breastfeeding can prevent the baby being overweight’, ‘It is advisable to breastfeed 3-4 times per day within 2-3 days after delivery’) still existed. The average scores for each item is shown in Table 2.

**Table 2:** The breastfeeding knowledge scores of pregnant women with GDM (*n* = 212)

Items	Scores (*M ± SD*, score)
1. Exclusive breastfeeding is the best option for baby up to 6 months of age.	4.85 ± 0.49
2. Containing antibody composition in breast milk, can enhance baby immunity and reduce disease occurrence.	4.86 ± 0.47
3. Early breastfeeding can prevent constipation of infants.	4.42 ± 0.84
4. Breastfeeding cannot prevent your baby from being overweight. ^a^	3.09 ± 1.25
5. Breastfeeding can reduce the incidence of allergic diseases in baby.	4.45 ± 0.86
6. Breastfeeding is conducive to the development of the baby’s intelligence.	4.49 ± 0.81
7. Breastfeeding is beneficial to uterine contraction, and can reduce postpartum hemorrhage.	4.64 ± 0.66
8. Breastfeeding cannot help the mother form an intimate relationship with the baby. ^a^	4.34 ± 1.32
9. Breastfeeding does not necessarily reduce a mother’s risk of developing breast cancer in the future. ^a^	3.96 ± 1.24
10. Breastfeeding does not necessarily reduce a mother’s risk of developing ovarian cancer in the future. ^a^	3.91 ± 1.18
11. Early and frequent sucking can promote milk secretion.	4.60 ± 0.75
12.The baby should be breast-fed within 2 h after delivery.	4.38 ± 0.90
13. It is advisable to breastfeed 3–4 times per day within 2–3 days after delivery. ^a^	3.18 ± 1.37
14.Breast milk can be frozen for 3 months after extrusion.	3.52 ± 1.12
15. Breast milk can keep fresh for 24–48 h in cold storage after extrusion.	3.56 ± 1.14
16. The breast milk can be microwaved before feeding the baby. ^a^	4.00 ± 1.14
17. Breastfeeding should be given according to the actual needs of the newborn.	4.45 ± 0.80
18. Although complementary foods are added after 4–6 months of age, breastfeeding can be maintained until the baby is 1–2 years old.	4.32 ± 0.89
19. Breast size affects milk production. ^a^	4.19 ± 0.97
20. Mothers with sunken nipples must not breastfeed. ^a^	4.30 ± 0.88
21. Mothers with cracked nipples must not breastfeed. ^a^	4.09 ± 1.02
22. After breastfeeding, the remaining milk should not be excreted. ^a^	4.06 ± 1.14
23. Water should be given to the baby after breastfeeding every time. ^a^	3.96 ± 1.21
24. Only 5-10 ml/ time should be fed to the baby on the first day after birth. ^a^	3.49 ± 1.10
25. Breastfeeding helps mothers regain their pre-pregnancy weight as quickly as possible.	4.40 ± 0.90
Total score	103.5 ± 10.4

Note: *GDM* Gestational diabetes mellitus; Breastfeeding benefits include item 2,3,4,5,6,7,8,9,10,25; Breastfeeding skills include item 1,11,12,13,17,22,23; Breast milk storage methods include 14,15,16; Breastfeeding conditions include item 18,19,20,21,24. ^a^Item 4, 8, 9, 10, 13, 16, 19, 20, 21, 22, 23, 24 were scored in reverse

### Univariate analysis of the factors for breastfeeding knowledge level

There were significant differences in breastfeeding knowledge level among GDM pregnant women with different characteristics. The scores of breast milk storage methods in different age groups were different (*F* = 3.234, *P* = 0.023). The overall score showed a downward trend with the increase of age. There were statistically significant differences between the groups with different educational level in total score, breastfeeding benefits, breastfeeding skills, breast milk storage methods and breastfeeding conditions (*P* < 0.05). This study revealed that the higher the family per capita monthly income, the higher its breastfeeding knowledge level in total score, breastfeeding benefits, breastfeeding skills, breast milk storage methods and breastfeeding conditions (*P* < 0.05). This study showed that pregnant women with GDM who have multiple knowledge sources had a higher level of breastfeeding knowledge than those who have single knowledge sources(*P* < 0.01). More detailed information is shown in Table 3.

**Table 3:** Subgroup analysis of breastfeeding knowledge level of pregnant women (*M ± SD*, score)

Subgroup	Breastfeeding benefits	Breastfeeding skills	Breast milk storage methods	Breastfeeding conditions	Total score
Age (year)					
≤ 29	42.3 ± 6.3	30.0 ± 3.9	11.5 ± 2.2	20.2 ± 3.4	104.0 ± 13.4
30–34	41.8 ± 6.5	29.5 ± 4.7	11.2 ± 2.3	20.6 ± 3.9	104.5 ± 9.9
35–39	42.4 ± 5.0	28.5 ± 3.3	10.9 ± 2.1	19.6 ± 2.9	101.4 ± 10.2
≥ 40	43.8 ± 5.1	29.9 ± 3.3	9.9 ± 1.6	21.1 ± 2.6	104.7 ± 8.1
*F*	0.740	1.500	3.234	1.838	1.361
*P* value	0.529	0.216	0.023	0.141	0.256
Educational level					
Junior High and below	37.6 ± 4.4	27.1 ± 3.8	10.0 ± 1.6	17.9 ± 2.8	92.6 ± 11.1
Senior High	38.5 ± 11.4	25.8 ± 8.8	10.1 ± 3.0	18.0 ± 5.8	98.9 ± 9.7
Junior college	41.9 ± 5.8	28.9 ± 3.6	10.7 ± 2.3	19.8 ± 3.1	101.3 ± 12.1
Bachelor or above	43.4 ± 4.6	30.1 ± 3.2	11.3 ± 2.0	21.0 ± 2.9	105.8 ± 8.8
*F*	7.463	7.825	3.394	7.528	10.075
*P* value	< 0.001	< 0.001	0.019	< 0.001	< 0.001
Nationality					
Han	42.5 ± 5.8	29.4 ± 4.0	11.0 ± 22.2	20.4 ± 3.3	103.9 ± 10.
Minority	39.7 ± 5.2	26.9 ± 4.3	10.6 ± 1.7	17.6 ± 3.9	94.7 ± 12.3
*t*	1.417	1.877	0.665	2.529	2.640
*P* value	0.158	0.062	0.507	0.012	0.009
Family per capita monthly income (yuan)					
< 3000	39.5 ± 6.4	27.4 ± 4.4	10.3 ± 2.2	18.6 ± 3.2	95.8 ± 14.3
3001–5000	41.0 ± 7.8	28.3 ± 5.5	10.6 ± 2.2	19.5 ± 4.0	101.3 ± 10.7
5001–10,000	42.3 ± 4.6	29.8 ± 3.3	11.2 ± 2.0	20.3 ± 3.1	103.5 ± 9.8
> 10,000	44.3 ± 4.6	30.1 ± 3.1	11.8 ± 2.2	21.6 ± 2.8	107.8 ± 8.0
*F*	4.298	3.312	3.870	5.393	7.103
*P* value	0.006	0.021	0.010	0.001	< 0.001
Husband’s education level					
Junior High and below	36.5 ± 13.1	25.7 ± 9.2	9.1 ± 3.3	16.4 ± 5.9	96.4 ± 11.6
Senior High	41.8 ± 5.5	30.3 ± 3.7	11.1 ± 1.9	19.9 ± 2.9	103.2 ± 11.4
Junior college	42.0 ± 5.5	28.8 ± 4.1	10.9 ± 2.1	19.9 ± 2.8	101.6 ± 12.2
Bachelor or above	43.1 ± 4.7	29.3 ± 3.3	11.2 ± 2.2	20.8 ± 3.4	104.7 ± 9.5
*F*	4.612	4.029	3.350	6.640	2.683
*P* value	0.014	0.008	0.020	< 0.001	0.048
Gestational age (week)					
≤ 36^+ 6^	40.3 ± 5.6	28.4 ± 4.1	10.5 ± 2.0	19.3 ± 3.0	98.5 ± 12.0
≥ 37	42.8 ± 5.8	29.6 ± 4.0	11.2 ± 2.2	20.5 ± 3.5	104.8 ± 9.7
*t*	−2.571	−1.732	−1.765	−2.201	−3.598
*P* value	0.011	0.085	0.079	0.029	< 0.001
Breastfeeding health education					
Yes	43.0 ± 6.4	29.8 ± 4.3	11.3 ± 2.4	20.7 ± 3.4	105.7 ± 9.8
No	41.4 ± 5.0	28.7 ± 3.7	10.8 ± 1.9	19.8 ± 3.5	100.7 ± 10.7
*t*	−2.100	−1.524	−1.574	−1.853	−3.335
*P* value	0.037	0.129	0.117	0.065	0.001
Number of knowledge source					
1	40.7 ± 7.0	28.4 ± 4.8	10.5 ± 2.3	19.3 ± 3.8	100.0 ± 11.5
≥ 2	43.5 ± 4.5	30.0 ± 3.3	11.4 ± 2.0	21.0 ± 2.9	105.9 ± 8.9
*t*	−3.562	−2.803	−3.243	− 3.768	−4.206
*P* value	< 0.001	0.006	0.001	< 0.001	< 0.001

### Multivariate analysis of factors for breastfeeding knowledge level

Multiple linear stepwise regression analysis was used to exploring the influence factors of breastfeeding knowledge level. This study found that gestational age, educational level, family per capita monthly income and multiple knowledge source were the independent protective factors for breastfeeding knowledge level, and minority nationality was the independent risk factor for breastfeeding knowledge level. The educational level had the greatest influence on the breastfeeding knowledge level of pregnant women with GDM (*β* = 0.210, *t* = 2.978, *P* = 0.003). The higher the education level of GDM pregnant women, the higher the breastfeeding knowledge level of GDM pregnant women. More detailed information is shown in Table 4.

**Table 4:** Multiple linear regression analysis of breastfeeding knowledge level

Variables	*β*	Standardized *β*	*t*	*P*
Constant	85.465	–	15.297	0.000
Gestational age (1 = gestational age < 36^+ 6^ week)	3.970	0.151	2.389	0.018
Nationality (1 = Han)	−7.942	−0.155	−2.478	0.014
Educational level (1 = Junior High and below)	2.471	0.210	2.978	0.003
Family per capita monthly income (1 = income < 3000 yuan)	1.784	0.151	2.196	0.029
Knowledge source (1 = 1 source)	1.407	0.172	2.661	0.008

Note:*R*^*2*^ = 0.226; Adjust *R*^2^ = 0.207; *F* = 4.948, *P* = 0.027. The value of Durbin Watson was 2.043, suggesting the independence of these variables. Tolerance in the model were as follows: gestational age 0.947, educational level 0.764, family per capita monthly income 0.808, and knowledge sources 0.908, nationality 0.976. “-” no value

## Discussion

This study investigated the breastfeeding knowledge level of pregnant women with GDM and explored its influence factors. The level of breastfeeding knowledge of women with GDM in this study was similar to that of normal pregnant women reported by other researcher [28]. However, some misconceptions of breastfeeding knowledge still existed in GDM population. Multiple linear regression analysis found that gestational age, family per capita monthly income, educational level, knowledge source were the independent protective factors for breastfeeding knowledge level and minority nationality was the independent risk factor for breastfeeding knowledge level. The educational level had the greatest influence on the breastfeeding knowledge level of GDM pregnant women.

This study showed that the 99.1% of GDM pregnant women had breastfeeding intention before delivery, which was higher than 73.2% reported by Dai and 92.17% reported by Zhang [29, 30]. It revealed that GDM pregnant women had a higher willingness to breastfeed. In Japan, researchers found that 96% of pregnant women expressed an intention to breastfeed. However, the breastfeeding rate was only 46% in 4 weeks postpartum. Although there was a strong desire to breastfeed before delivery, pregnant women may stop breastfeeding when they encounter difficulties or obstacles that are difficult to solve in postpartum. Therefore, it suggested that nurses or midwifery should pay more attention to GDM pregnant women who have breastfeeding intention and strengthen their intention by health education or peer education. Additionally, nurses or midwifery should focus on postnatal breastfeeding status of GDM women and assisted them to solve the difficulties to breastfeeding in a timely manner. Moreover, to form correct breastfeeding cognition and improve breastfeeding rate of women with GDM, scientific and systematic health education should be carried on these population.

This study showed that pregnant women with GDM had an average level of breastfeeding knowledge. However, some misconceptions of breastfeeding knowledge still existed in GDM population. Pregnant women with different sociodemographic characteristics had different understanding degree of breastfeeding knowledge. GDM pregnant women who did not receive health education of breastfeeding had insufficient understanding of breastfeeding conditions, such as ‘breast size affecting milk secretion’. However, GDM pregnant women who received health education on breastfeeding had significantly higher knowledge level, which was consistent with the conclusion of Thomas’s report [31]. GDM pregnant women who had lower educational level, premature birth, and lower family per capita income, and whose husband had lower educational level were not optimistic about the mastery of breastfeeding knowledge about breastfeeding conditions and breastfeeding benefits, which was supported by some researchers’ reports [20, 23, 32]. Research revealed that the higher the knowledge level of breastfeeding, the less likely it is to terminate breastfeeding early [33]. Breastfeeding knowledge education can help avoid the occurrence of early interruption of breastfeeding, suggesting that breastfeeding knowledge education was necessary for GDM pregnant women.

Multivariate regression analysis revealed that gestational age, education level, family per capita monthly income, and knowledge source were independent protective factor for breastfeeding knowledge level. This result was similar to Thomas’ study [31]. The study found that breastfeeding counseling, socioeconomic status and educational level had a great impact on breastfeeding cognition of primipara. However, Thomas’s study did not report the impact of gestational age and knowledge source on breastfeeding cognitions. This study showed that the education level had the greatest influence on the knowledge of breastfeeding. Therefore, in nursing work, pregnant women with lower educational level cannot be ignored. These population had weaker ability to understand and grasp the knowledge. Therefore, there was a need for detailed and in-depth health education on breastfeeding for low-educational-level pregnant women. There was a growing concern among pregnant women about the breastfeeding knowledge with the increasing of gestational age. Study showed that 66.3% of pregnant women were willing to receive knowledge education in the second trimester [20], which was the time for pregnant women to have regular prenatal examination. Therefore, medical staff should make full use of this period to educate pregnant women about breastfeeding. Moreover, studies found that the medical staff is the main source of breastfeeding knowledge of women and is also the main object for help of any difficulty of women. In some countries and regions, pregnant women get knowledge mainly by means of the media and women’s magazines [20, 33]. Therefore, GDM pregnant women should be provided health education on breastfeeding through a variety of ways, which is conducive to improving the knowledge level of breastfeeding. More diversified forms of health education should be carried out, such as breastfeeding salons and WeChat network platforms, and Internet media resources should be fully utilized to carry out health education.

This study had three limitation. First, only one hospital was included in this study, which is the one of the best hospitals of west China. Our participants almost were well educated and had high household income. So, this may reduce the representativeness of the sample in this study. Second, Multiple linear regression analysis showed that the influencing factors studied in this study could only explain 20.7% of the variation in breastfeeding knowledge score, revealing that there were other factors that did not be found and need to be studied. Thirdly, the validation and pilot-testing of self-designed questionnaire were not conducted.

## Conclusion

A cross-sectional survey was conducted in West China. This study showed that GDM pregnant women had a strong willingness to breastfeed before delivery, and an average level of breastfeeding knowledge. However, some misconceptions of breastfeeding knowledge still existed in these GDM population. Pregnant women with GDM and gestational age less than 37 weeks of gestation, low educational level, low family per capita monthly income and single access to knowledge should be the focus of health education on breastfeeding. Therefore, special attention should be paid to these population. Systematic and in-depth breastfeeding health education should be carried on these women. Medical staff were still the main source of breastfeeding knowledge for pregnant women. Thus, medical staff especially nurse and midwifery should play a major role in breastfeeding health education. At the same time, more diversified forms of health education, such as breastfeeding salons, WeChat network platforms, and Internet media resources should be fully utilized to carry out health education for pregnant women with GDM.

## Supplementary Information

**Additional file 1.** Questionnaire on breastfeeding knowledge and its influence factors of pregnant women with gestational diabetes mellitus.

## Data Availability

The datasets used and/or analyzed during the current study are available from the corresponding author on reasonable request.

## Abbreviations

GDM: Gestational diabetes mellitus
WHO: World Health Organization
UNICEF: United Nation Children’s Fund
M ± SD: Mean ± Standard Deviation
OGTT: Oral glucose tolerance test
