# Supplementary material for: Exploring the breastfeeding knowledge level and its influencing factors of pregnant women with gestational diabetes mellitus
Source: BMC Pregnancy Childbirth. 2020 Nov 23;20:723. doi: 10.1186/s12884-020-03430-9 (PMC7685611; doi:10.1186/s12884-020-03430-9)
Supplement: Supplementary file 1 — Additional file 1. Questionnaire on breastfeeding knowledge and its influence factors of pregnant women with gestational diabetes mellitus. [file 12884_2020_3430_MOESM1_ESM.docx]

**Questionnaire on breastfeeding knowledge and its influence factors of pregnant women with gestational diabetes mellitus**

1. How old are you? ___________years old.

2. What’s your weight? ___________kg.

3. What’s your height? ___________cm.

4. What’s your gestational age? ___________weeks.

5. Delivery times? ___________

6. Educational level (single topic selection)

1) Primary school and below; 2) Junior high school

3)Senior high school and technical secondary school; 4) Junior college

5) Bachelor or above

7. Nationality (single topic selection)

1) Han nationality; 2) Zang nationality; 3) Yi nationality; 4) Hui nationality

5) Others minority nationality (Please note___________)

8. Occupation

1) Professional; 2) Administrative; 3) Clerk; 4) Farmer; 5) Freelance; 6) Unemployed

9. Home address:

1) Chengdu city; 2) second-tier city; 3) county; 4) township; 5) countryside

10. Marital status:

1) Never married; 2) Married; 3) Divorced; 4) Widowed

11. Family per capita monthly income (yuan)

1) <3000

2) 3001-5000

3) 5001-10000

4) >10000

12. Do you smoke?

1) Yes (if your answer is ‘Yes’, please turn to 12-1); 2) No

12-1The number of cigarettes per day ___________

13. Main caregivers in your pregnancy:

1) Self; 2) Husband; 3) Mother; 4) Mother-in-law; 5) Nanny; 6) Relatives

14. Husband’s educational level:

1) Primary school and below; 2) Junior high school

3)Senior high school and technical secondary school; 4) Junior college; 5) Bachelor or above

15. Husband’s occupation

1) Professional; 2) Administrative; 3) Clerk; 4) Farmer; 5) Freelance; 6) Unemployed

16. Breastfeeding knowledge source:

1) Book; 2) Newspaper/magazine; 3) Internet; 4) Television programs; 5) Family member or friends; 6) Medical staff

17. How many times you participated in breastfeeding health education during pregnancy? ___________times.

18. Did you have breast surgery?

1) Yes; 2) No

19. What’s the status of your nipples at rest?

1) Prominent; 2) Flat; 3) Sunken

20. Did you have breastfeeding experience?

1) Yes (if your answer is ‘Yes’, please turn to 20-1); 2) No

20-1. How long did you breastfeed in your last breastfeeding experience? ___________months.

21. Do you have breastfeeding intention?

1) Yes (if your answer is ‘Yes”, please turn to 21-1); 2) No

21-1. How long do you plan to breastfeed in this time? ___________months.
